# Distribution characteristics of organic carbon (nitrogen) content, cation exchange capacity, and specific surface area in different soil particle sizes

**DOI:** 10.1038/s41598-023-38646-0

**Published:** 2023-07-28

**Authors:** Xiaoqian Bi, Hang Chu, Mingming Fu, Dandan Xu, Wenyu Zhao, Yijian Zhong, Mei Wang, Ke Li, Ya-nan Zhang

**Affiliations:** 1 Collaborative Innovation Center for Water Pollution Control and Water Safety in Karst Area, Guilin University of Technology, Guilin, 541004 China; 2 The Guangxi Key Laboratory of Theory and Technology for Environmental Pollution Control, Guilin University of Technology, Guilin, 541004 China; 3 Hengsheng Water Environment Treatment Co., Ltd., Guilin, 541100 China; 4 College of Civil Engineering and Architecture, Guilin University of Technology, Guilin, 541004 China; 5 College of Environmental Science and Engineering, Guilin University of Technology, Jiangan Road 12, Guilin, 541004 Guangxi China

**Keywords:** Environmental sciences, Environmental social sciences

## Abstract

Understanding the distribution of soil organic carbon and nitrogen (OC(N)) content, cation exchange capacity (CEC), and specific surface area (SSA) in different soil particle sizes is crucial for studying soil fertility and properties. In this study, we investigated the distribution characteristics of the OC(N), CECand SSA in different particles of yellow–brown soil under different methods. The result revealed that as the particle size decreased, the soil OC(N), SSA and CEC content gradually increase. The content of OC and ON different soil particles ranged from 1.50–28.16 g·kg^−1^ to 0.18–3.78 g·kg^−1^, respectively, and exhibited significant differences between different particles. We observed good linear relationships between OC and ON in different particle sizes of yellow–brown soil under different utilization methods, with correlation coefficients ranging from 0.86 to 0.98, reaching a very significant level (n = 12, *p* < 0.01). The ranges of SSA and CEC in different particles of the four soils were 0.30–94.70 m^2^·g^−1^ and 0.70–62.91 cmol·kg^−1^, respectively. Additionally, we found logarithmic relationships between SSA (CEC) and the equivalent diameter for the four soils, with correlation coefficients (r^2^) higher than 0.91. Furthermore, there was an extremely significant linear relationship between CEC and SSA of the four soils, with correlation coefficients (r^2^) of 0.92–0.97 (n = 12, *p* < 0.01). These results highlight the close relationship between soil particle size and soil OC(N), SSA, and CEC. The conclusions drawn from this study provide valuable data support and a theoretical basis for further understanding soil properties.

## Introduction

Soil, as a heterogeneous body, consists of minerals derived from rock weathering, organic matter from plant, animal, and microbial residues, soil organisms, soil water, and soil air. The physical and chemical properties of soil can vary significantly due to the diversity of soil particles in terms of type and size. Recently, with the emergence of the "double carbon" plan, research on carbon sequestration has gained momentum. It includes investigations into the accumulation and preservation of organic matter in the soil^1–3^, dynamic changes in organic matter content^4–6^, and the potential of soil to offset increasing atmospheric CO_2_^7^. Soil carbon sequestration offers an opportunity to enhance soil organic carbon content, contribute to global food security, improve public health, and serve as a strategy to mitigate and adapt to climate change.

The interaction between soil organic carbon (OC) and soil minerals depends not only on the physical and chemical properties of mineral components but also on the morphology and chemical structure of organic carbon^8–13^. Soil mineral surface adsorption plays a critical role in the preservation of soil organic carbon. However, the ability of soil organic carbon accumulation is not solely determined by the specific surface area (SSA)^14,15^, but is also influenced by factors such as soil particle size and shape^16,17^, surface roughness^18,19^, and the inner surface of the dilatability minerals^20^. Soil particle size is a major physical property that determines or affects soil strength, nutrient status, soil erosion, etc. Soil particle size distribution is also an indicator of the influence of different land use methods on soil properties such as OC(N), SSA and CEC. The information on clay-OC micro aggregates can protect soil OC, and the surface area of these aggregates is associated with the soil adsorption process of the soil^21,22^. As SOC adsorbs and desorbs soil agglomerates, the agglomerates will continue to develop and break down. When SOC is artificially added to the soil, organic matter accumulates more in large agglomerates but decomposes more rapidly than in micro-agglomerates^23^. Studies have shown that the stability of organic matter in soil agglomerates may be related to soil use patterns. The surface soil aggregates of silvicultural soils were more stable than those of pasture soils^24^. Soil cation exchange capacity (CEC) is an important parameter that describes the adsorptive characteristics of soil^25–28^, and it is influenced by the negative charges on the surface of the clay minerals and the OC^29^. With the increase in pH, the amount of variable negative charge in organic soil increases, and CEC also increases^27,30^.

However, the relationship between SSA, CEC, and soil organic carbon content has been widely recognized, but their relationship with soil micro aggregates of different diameters is less understood. Additionally, the nitrogen in the soil is primarily present as organic nitrogen in soil organic matter. Studies have shown a significantly positively correlated between the total nitrogen content and the organic carbon content of the soil^31^, as well as between the soil carbon–nitrogen ratio and organic matter content^32^. Besides, the relationship between soil CEC and SSA and soil organic nitrogen content remains less well-known. This study focuses on four yellow–brown soils with different utilization methods as the research object. Specifically, we investigate (1) the contents of OC(N), SSA, and CEC in different soil layers and particle sizes, and (2) the detailed relationships between OC and ON, soil equivalent diameter, and SSA or CEC, OC(N) and SSA or CEC, as well as SSA and CEC. This study investigates the soil aggregate particle size distribution, organic matter content, specific surface area, and cation exchange capacity of four different land use types of yellow–brown soil in Xinyang City, Henan Province, China. The aim is to elucidate the soil fertility potential and reveal the distribution characteristics of soil aggregate particle size, organic matter content, specific surface area, and cation exchange capacity under different land use types of yellow–brown soil. This research provides valuable data support and a theoretical basis for further understanding soil properties.

## Materials and methods

### Test soil

The tested soils were collected on 10 July 2022 from yellow–brown soil with four different utilizations in Xinyang, Henan Province, including paddy soil (32 624 N, 114°9´37 E), woodland soil (32 0151 N, 114 451 E), dryland cultivated soil (31 4921 N, 115 0145 E) and vegetable garden soil (32 08′ 09 "N, 114 05′ 46"E), in which three sections (0–10 cm, 10–20 cm, and 20–30 cm) were collected for each soil, and their basic physical and chemical properties are shown in Table 1.

**Table 1:** Selected properties of yellow–brown soils.

Soil	Depth/cm	pH	CEC	SSA/m^2^·g^−1^	Particle size composition of micro aggregates			
					Sand	silt	Coarse clay	Fine clay
					20–250 μm	2–20 μm	0.2–2 μm	< 0.2 μm
					/%	/%	/%	/%
Vegetable garden soil (VG soil)	0–10	6.67	21.48	29.9	34.4	33.6	24.8	7.2
	10–20	6.63	19.62	28.9	38.8	32.7	23.3	5.2
	20–30	6.52	20.78	34.2	34.3	33.2	27.4	5.1
Paddy soil (Py soil)	0–10	5.81	12.93	19.5	36	42.5	16.6	4.9
	10–20	6.24	14.02	19.7	34.3	43.7	18.6	3.4
	20–30	6.86	12.37	23.2	43.6	36.4	14.9	5.1
Woodland soil (WL soil)	0–10	6.37	17.08	19.5	56.9	21.2	18.3	3.6
	10–20	5.87	16.92	26.9	42.5	31.6	23	2.9
	20–30	6.03	15.92	28.1	39.6	32.5	23.2	4.7
Dryland cultivated soil (DL soil)	0–10	5.95	9.64	9.1	64.6	21.8	11.4	2.2
	10–20	5.53	9.11	11.3	69.6	15	12.1	3.3
	20–30	5.85	8.96	8.2	70.8	11.7	15.2	2.3

### Research method

#### Classification of soil micro-aggregates

Dispersion of soil samples According to the method of literature^33^, weigh the soil samples with a diameter greater than 2 mm, make a soil suspension with a soil-to-water ratio of 1:5, disperse the suspension by ultrasonic wave, and pass it through a 250 μm sieve and a 20 μm sieve by wet sieve method. Those that pass through the 20 μm sieve have a particle size of < 20 μm, and those that do not pass through the 20 μm sieve have a particle size of 20–250 μm (sand part),According to the sedimentation method, the sedimentation time of < 2 μm particles at 10 cm is obtained according to the temperature look-up table. Gently insert the siphon into the beaker 30 s before the specified liquid suction, siphon the colloidal suspension to another container, and repeatedly siphon until the suspension is no longer turbid within the specified sedimentation time^34^, and the sedimentation part belongs to the 2–20 μm part (powder part),Particles less than 2 μm are centrifuged at 4100 r/min for 16 min, the suspension is collected, and the centrifugation process is repeated until the suspension is clear so that the particle size of < 0.2 μm (fine clay particles) is obtained, and the remaining soil samples are 0.2–2 μm (coarse clay particles). Samples of each particle size are dried at 40 °C, sieved by 100 mesh sieve, and stored in the refrigerator for later use.

#### Testing of soil samples

Determination of soil organic carbon and nitrogen with Macro Elemental Analyzer (Vario MAX CN, Elementar) for soil. The SSA is determined by the N2 adsorption method, and the instrument is a fully-automatic SSA and pore size analyzer F-Sorb3400 (Beijing Kinai spectrum). The cation substitution amount is determined by the ammonium acetate method with pH 7.0.

### Calculation and statistical analysis

The statistical data were analyzed by variance with DPS software, and the significance of differences between treatments was tested by LSD. Graphpad Prism 4.0 software was used for mapping and regression analysis.

The relative proportion of SSA of each particle size is defined as the ratio of the number of SSAs of each particle size of micro-aggregate to the sum of the number of SSAs of each particle size of micro-aggregate.

## Result and discussion

### Changes of OC(N) content in yellow–brown soil

The carbon sequestration effect of soil aggregates is closely related to the protection mechanism of aggregates^35,36^. With the in-depth study of soil carbon sequestration, people gradually realize that soil aggregates are not only an important index of soil fertility^37,38^ but also a carrier of soil organic carbon stabilization and protection. Changes in OC(N) content in yellow–brown soil with grain size were shown in Table 2. From the results of this experiment, the content of OC in yellow–brown soil ranged from 1.50 to 28.16 g·kg^−1^, and that of ON ranged from 0.18 to 3.78 g·kg^−1^. The contents of soil OC and ON are mainly concentrated in clay soil less than 2 μm, followed by aggregates 2–20 μm, and finally 20–250 μm. The contents of OC(N) in yellow–brown soil in each soil layer increase with the grain size decreasing. Through variance analysis and multiple comparisons of different soil layers, there were differences in OC(N) contents of yellow–brown soil with different utilization patterns among different grain grades. It can be seen that soil OC and ON contents are affected by particle sizes. It has been reported that there is a significant positive correlation between soil OC content and clay content, which is used to illustrate the influence of clay protection on soil OC storage potential^39,40^. Zhou et al.^41^ studied that the physical protection of aggregate particles is the main mechanism of newly added OC accumulation in several typical paddy soils in South China under good cultivation and fertilization, especially in red paddy soils. Tan et al.^42^ studied the distribution of OC in aggregates under different land use patterns and found that 69.8–86.6% of OC was distributed in micro-aggregates with a particle size of less than 50 μm, among which 2–20 μm micro-aggregates had the largest proportion of OC, and micro-aggregates had strong carbon fixation ability. Li et al.^43^ showed that the OC in paddy soil of red soil decreased with the increase of soil particle size, in which the OC of < 2 μm and 2–20 μm accounted for 29.2% and 30.7% of the total OC, respectively. Therefore, soil microstructure plays an important role in protecting soil OC, especially the content of soil clay plays a direct and indirect role in protecting soil OC.

**Table 2:** The change of OC(N) along with particles of yellow–brown soils.

Soil layer/cm	Particle size/μm	VG soil	Py soil	WL soil	DL soil	Significant difference between particles	VG soil	Py soil	WL soil	DL soil	Significant difference between particles	VG soil	Py soil	WL soil	DL soil	significant difference between particles
		C total/g·kg^−1^					N total/g·kg^−1^					C/N				
0–10	20–250	11.82	3.65	6.17	4.49	Cc	0.61	0.25	0.45	0.46	Cc	19.38	14.6	13.71	9.76	Aa
	2–20	25.34	12.93	15.41	5.28	Bb	2.53	1.21	1.51	0.53	Bb	10.02	10.69	10.21	9.96	ABa
	0.2–2	25.29	21.62	23.99	15.94	Aa	3.64	2.63	3.29	2.09	Aa	6.95	8.22	7.29	7.63	Bb
	< 0.2	28.16	22.65	27.75	17.31	Aa	3.4	3.23	3.78	2.34	Aa	8.28	7.01	7.34	7.4	Bb
10–20	20–250	8.21	3.91	1.74	4.11	Bc	0.44	0.27	0.21	0.42	Cc	18.66	14.48	8.29	9.79	Aa
	2–20	21.87	15.03	12.1	4.74	Ab	2.19	1.51	1.31	0.49	Bb	9.99	9.95	9.24	9.67	ABa
	0.2–2	18.68	20.54	14.68	13.36	Aa	2.57	2.65	2.08	1.85	Aa	7.27	7.75	7.06	7.22	Bb
	< 0.2	21.33	21.69	17.76	13.52	Aa	3.03	3.03	2.48	1.89	Aa	7.04	7.16	7.16	7.15	Bb
20–30	20–250	2.72	1.9	1.5	2.42	Bc	0.2	0.18	0.19	0.24	Cc	13.6	10.56	7.89	10.08	Aa
	2–20	13.25	7.89	7.37	7.97	Ab	1.45	0.82	0.9	0.82	Bb	9.14	9.62	8.19	9.72	ABa
	0.2–2	13.26	16.55	11.93	8.96	Aa	1.77	2	1.76	1.33	Aa	7.49	8.28	6.78	6.74	Bb
	< 0.2	15.84	13.64	14.89	9.31	Aa	2.11	2.19	2.1	1.39	Aa	7.51	6.23	7.09	6.7	Bb

*Different capital letters represent significant differences (*p* < 0.01) and different lowercase letters represent significant differences (*p* < 0.05) between different particle sizes in the same soil layer.

As the particle size becomes finer, the soil C/N generally decreases gradually, with the range of 7.89–19.38 for 20–250 μm and 8.19–10.69 for 2–20 μm and 6.23–8.28 for < 2 μm particles. The C/N ratios of > 2 μm two particles were significantly different from that of particles less than 2 μm two particles. The C/N ratio in clay is low, which is a typical feature of organic-mineral aggregates to stabilize organic matter^44^.

**Figure 1:**
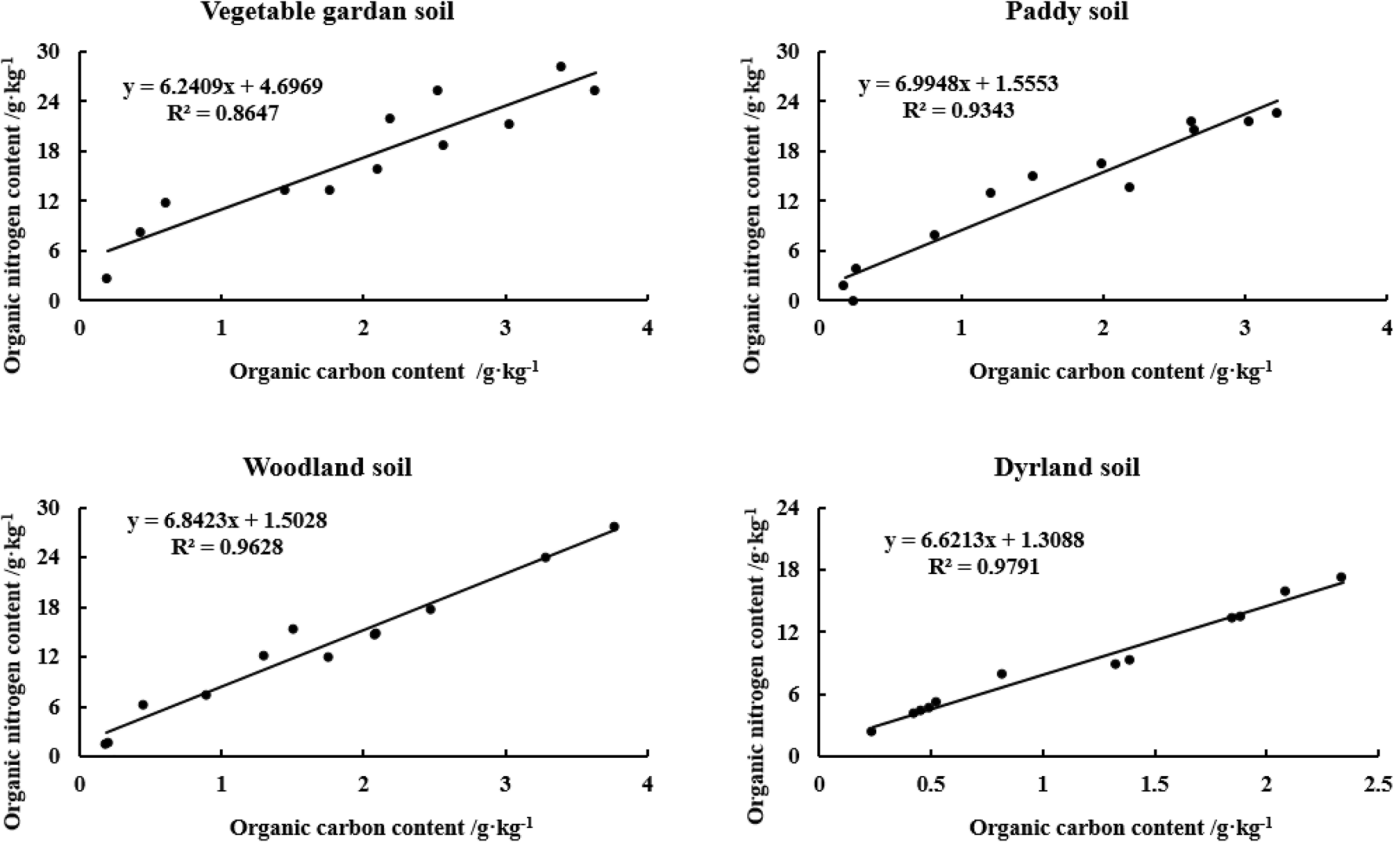
Correlation between OC and ON in different particle sizes of yellow–brown soil under different.

There were good linear relationships between OC and ON in different particle sizes of yellow–brown soil under different utilizations, with correlation coefficients of 0.8647, 0.9343, 0.9628, and 0.9791, respectively, and the linear relationship reached a very significant level (n = 12, *p* < 0.01) (Fig. 1).

**Figure 2:**
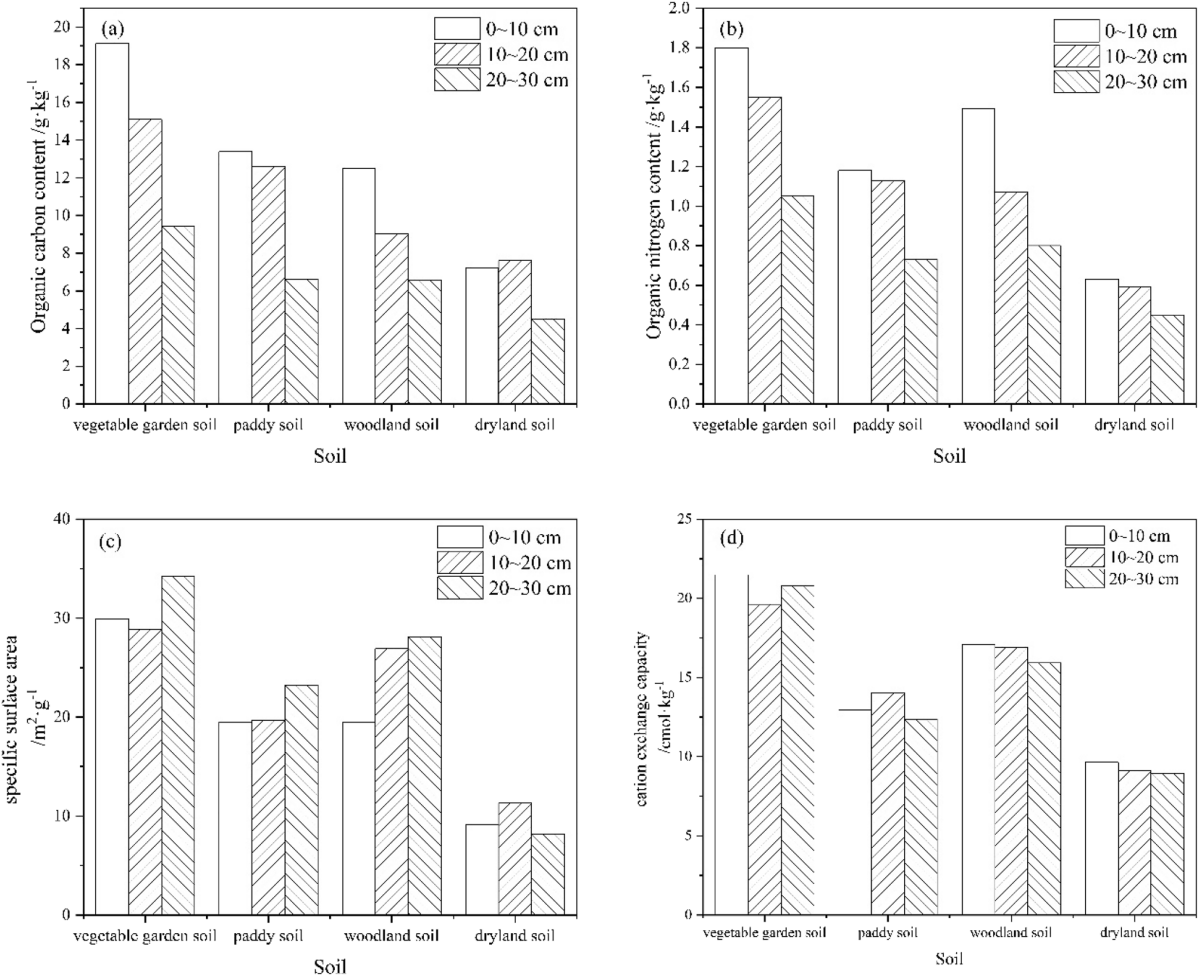
Distribution characteristics of OC,ON,SSA,CEC in different soil layers of four different types of yellow–brown soils.

For different soil layers of yellow–brown soil, the OC(N) contents in three soil layers, are gradually decreasing with the deepening of the four soils (Table 3). In this study, the content of soil organic carbon and organic nitrogen showed a decreasing trend with increasing soil depth in different soil layers of the profile (Fig. 2a,b), the result was similar to Mishra and Gorai^45^. The influence of soil layer on the content of OC(N) is not significant between 0–10 cm and 10–20 cm, but extremely significant with 20–30 cm. On the one hand, it was related to the dead fallen matter, as most of the dead leaves of soil ground vegetation were distributed on the ground, and a large number of dead leaves decomposed or formed humus, releasing a large number of nutrients into the soil, making the organic carbon and nitrogen of soil 0–20 cm higher than other soil layers. On the other hand, such as in vegetable garden land and rice field, the upper soil layer is mainly affected by management measures such as artificial seed turning, fertilization, and weeding. Over time, organic carbon and nitrogen from exogenous sources migrate from the upper layer to the lower layer, and the influence diminishes with increasing depth, making the organic carbon and nitrogen content of the upper soil layer higher than that of the lower soil layer. The rice field is generally in a flooded state, and the migration of organic carbon and nitrogen in the rice field soil is a leaching transfer. Organic carbon and nitrogen may migrate with the vertical infiltration of seepage water, which also means that high-intensity artificial disturbance is very likely to cause carbon and nitrogen loss in soil.

**Table 3:** The changes of OC(N) content along with soil depth by different utilizations of yellow–brown soil.

Soil layer/cm	VG	Py	WL	DL	significant difference between soil layers	VG	Py	WL	DL	Significant difference between soil layers
	soil	soil	soil	soil		soil	soil	soil	soil	
	C/g·kg^−1^					N/g·kg^−1^				
0–10	19.12	13.4	12.5	7.2	Aa	1.8	1.18	1.49	0.63	Aa
10–20	15.1	12.6	9.03	7.6	Aa	1.55	1.13	1.07	0.59	ABa
20–30	9.45	6.62	6.55	4.5	Bb	1.05	0.73	0.8	0.45	Bb
Utilizations	Aa	ABb	Bbc	Bc	–	Aa	Bb	ABb	Cc	–

*Different capital letters represent significant differences (*p* < 0.01) and different lowercase letters represent significant differences (*p* < 0.05) between different soil layers and among soils with different utilizations.

In this study, for different utilization methods of yellow–brown soil, the order of OC(N) content is vegetable garden soil > paddy soil > woodland soil > dryland soil. And the OC(N) content of vegetable garden soil is significantly different between paddy soil, woodland soil, and dryland soil (*p* < 0.05). Firstly, the organic carbon and nitrogen of the soil were influenced by climate as well as human and other factors. The organic carbon and nitrogen content of vegetable garden soil were significantly higher than the other three types of soil. In general, vegetable garden soils were tilled and fertilized with organic fertilizers all year round, which facilitated the surface concentration of organic carbon and nitrogen in the soil. Similarly, as a rice field, fertilization is one of the reasons for increasing the organic nitrogen content. At the same time, fertilizer application also promotes the growth of the root, stem, and leaf systems of crops, which has a certain loosening effect on the upper layer of soil, thus promoting the migration of organic carbon and nitrogen. The straw and roots are decomposed in a moist environment, effectively increasing the organic matter content in the soil of rice fields. Woodland soils are mostly alpine and have a pronounced annual herbaceous vegetation. Due to the wet and cold climatic conditions, the withered herbaceous plants are easily decomposed by the biotope to form nutrient accumulation. Coupled with more natural apoplastic material in woodland, a large amount of organic matter is formed in the topsoil under the action of microbial decomposition. In contrast, Dryland soils (such as peanut and corn fields), on the other hand, are not fertilized as much as vegetable and rice fields, and the above-ground portion of vegetation after harvest, which is generally recycled, rarely returns to the soil surface. Compared to forest land soils, the number of dead branches and fallen leaves returned to dry farmland was significantly reduced, and the organic carbon and nitrogen content of the surface layer (0–20 cm) soil was significantly lower compared to forest land.

The reason for the high content of soil OC and ON in vegetable fields may be related to the application of organic fertilizers for many years. Secondly, the paddy soil of paddy-upland rotation is favorable for the accumulation of OC under the condition of flooding. The contents of OC and ON in the surface layer of woodland soil in Sang Yu, which has been growing for many years, are higher mainly related to the decomposition of litter. The content of OC and ON in dryland peanut fields in the least because of the sandy soil. Therefore, it is important to improve soil management measures, reasonably increase organic fertilizers, maintain soil fertility, and avoid unreasonable soil use in terms of carbon and nitrogen fixation considerations.

### Changes of SSA in yellow–brown soil

**Figure 3:**
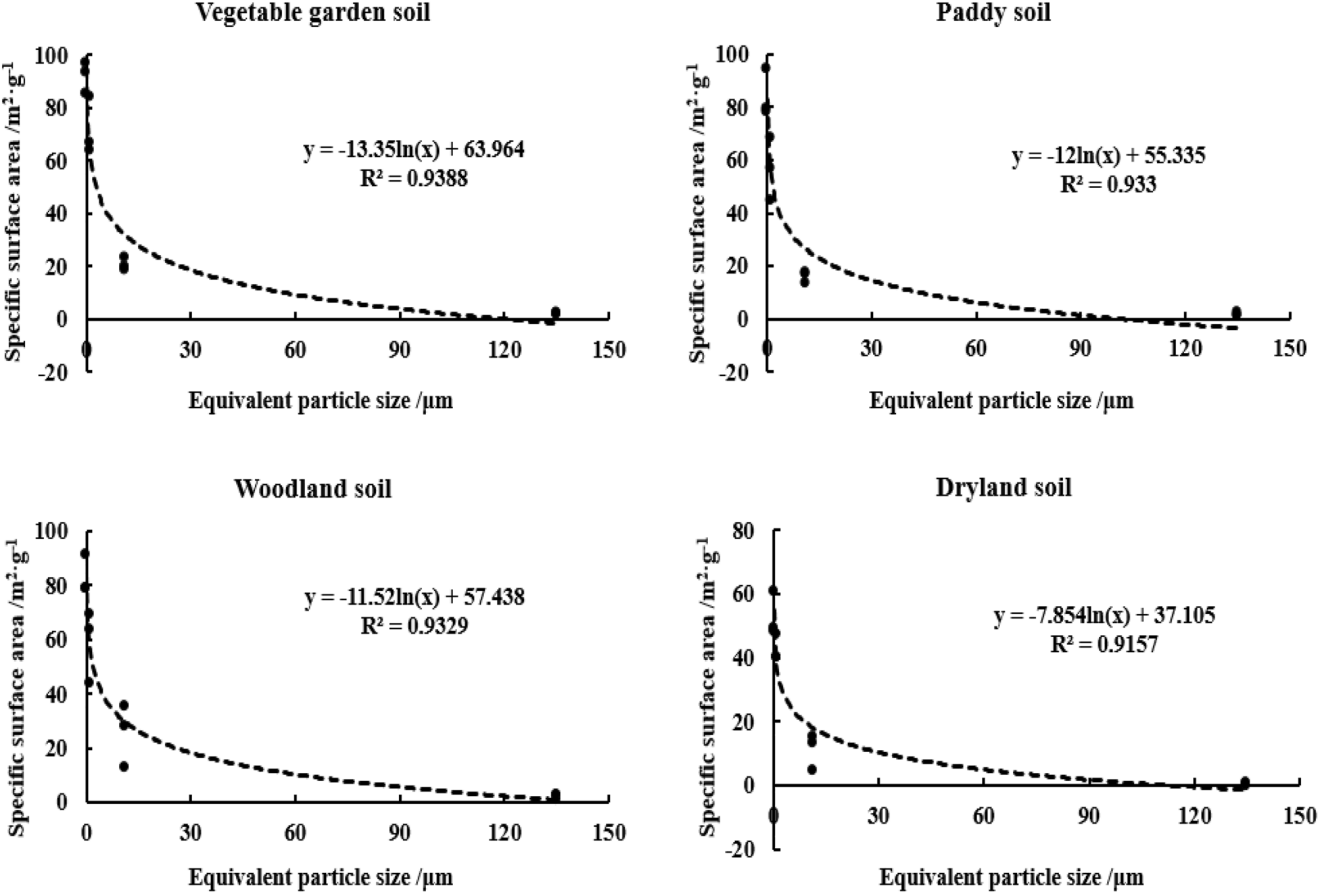
Correlation between SSA and equivalent particle size in yellow–brown soil under different utilizations.

The change of SSA in yellow–brown soil is shown in Table 4. The SSA ranges from 0.3–23.9 m^2^·g^−1^ at two particle sizes larger than 2 μm, and 40.6–94.7 m^2^·g^−1^ at the two particle sizes less than 2 μm. As the particle size becomes finer, the SSA increases, which was in a logarithmic relationship with the equivalent diameter for the four soils with the correlation coefficients (r^2^) of 0.9388, 0.9330, 0.9329 and 0.9157, respectively (n = 12, *p* < 0.01) (Fig. 3). Through the analysis of variance and multiple comparisons, the SSA content of yellow–brown soil with 20–250 μm particles is not significantly different from that of 2–20 μm particles, but it is significantly different from that of 0.2–2 μm and < 0.2 μm particles and the difference between the two clay particles is significant or extremely significant. However, the SSA relative scale of the four soils in different particle sizes showed that 0.2–2 μm (38.3–65.2%) > 2–20 μm (10.4–39.4%) > less than 0.2 μm (10.1–22.1%) > 20–250 μm (2.6–8.2%) particles. In the yellow–brown soil with four different utilizations, although the SSA of clay particles was the largest, the content of clay particles in the soil was relatively low, resulting in the SSA relatively low proportion. Similarly, although the SSA of the 2-20 μm particles was not very high, the particles high content in the soil, resulted in the SSA relatively higher proportion.

**Table 4:** Change of SSA in yellow–brown soil.

Soil layer/cm	Particle size/μm	Equivalent diameter/μm	VG soil		Py soil		WL soil		DL soil		Significant difference between particles
			SSA/m^2^·g^−1^	Relative scale/%	SSA/m^2^·g^−1^	Relative scale/%	SSA/m^2^·g^−1^	Relative scale/%	SSA/m^2^·g^−1^	Relative scale/%	
0–10	20–250	135	2.4	2.6	1.9	3.5	1.6	3.9	0.7	4.7	Cc
	2–20	11	23.9	25.3	17.8	38.4	36	33.0	15.6	35.5	Cc
	0.2–2	1.1	64	50.0	45.4	38.3	64	50.7	40.7	48.5	Bb
	< 0.2	0.1	97.5	22.1	79.8	19.9	79.3	12.4	48.8	11.2	Aa
10–20	20–250	135	2.5	3.6	1.7	2.9	3.2	6.0	0.3	2.8	Bc
	2–20	11	19.1	22.9	14.1	30.6	28.3	39.4	5.2	10.4	Bc
	0.2–2	1.1	67.1	57.2	57.5	53.2	44	44.5	40.6	65.2	Ab
	< 0.2	0.1	85.9	16.4	78.7	13.3	79.3	10.1	49.4	21.6	Aa
20–30	20–250	135	2.9	2.8	2.6	5.0	2.8	4.3	1.3	8.2	Cd
	2–20	11	20	18.7	17.3	27.9	13.2	16.6	13.8	14.4	Cc
	0.2–2	1.1	84.3	65.0	69.1	45.6	69.8	62.5	47.7	64.8	Bb
	< 0.2	0.1	93..9	13.5	94.7	21.4	91.6	16.6	61.1	12.6	Aa

*Different capital letters represent significant differences (*p* < 0.01) and different lowercase letters represent significant differences (*p* < 0.05) between different soil layers and among soils with different utilizations.

In terms of soil layer distribution, the SSA of the soil increases with increasing depth of the soil layer (Fig. 2c). In general, soil properties gradually change from large granular to fine granular from the top to the bottom of the soil layer. However, vegetable garden soils have high organic matter content due to the surface (0–10 cm) planting layer being tilled and fertilized or sprayed with herbicides all year round, resulting in loose and finer soil particles with large specific surface area; secondly, the middle (10–20 cm) soil layer is subjected to frequent tillage and disturbance, which may lead to uneven distribution of organic matter in this soil layer. The areas with lower organic matter content may lead to the aggregation of larger particles and thus a slightly smaller specific surface area. The lower layer is less tilled and disturbed, and the surface area of particles is relatively compact and fine, with a sudden increase in the specific surface area of the soil. Rice fields, due to their use, are generally cultivated in the 0–20 cm layer and are flooded for a long time, resulting in uniform soil particles in the 0–20 cm layer and thus little difference in the specific surface area of the 0–20 cm soil layer. In contrast, woodland soils are rarely disturbed by humans, and there exists a more stable soil structure and particle arrangement; in addition, vegetation cover can also reduce soil erosion and particle loss, which is conducive to maintaining a higher specific surface area. Therefore, the distribution characteristics of its specific surface area in different soil layers are vertically distributed and increase with the deepening of soil layers. The possible explanations for the sudden decrease of specific surface area in the lower layer of the dry tillage are, as mentioned earlier, the significant decrease of organic carbon and nitrogen content, which leads to the aggregation of larger particles and thus a smaller specific surface area; on the other hand, the low water content may cause the soil to be easily sandy and the specific surface area also becomes smaller.

### Changes of CEC content in yellow–brown soil

**Figure 4:**
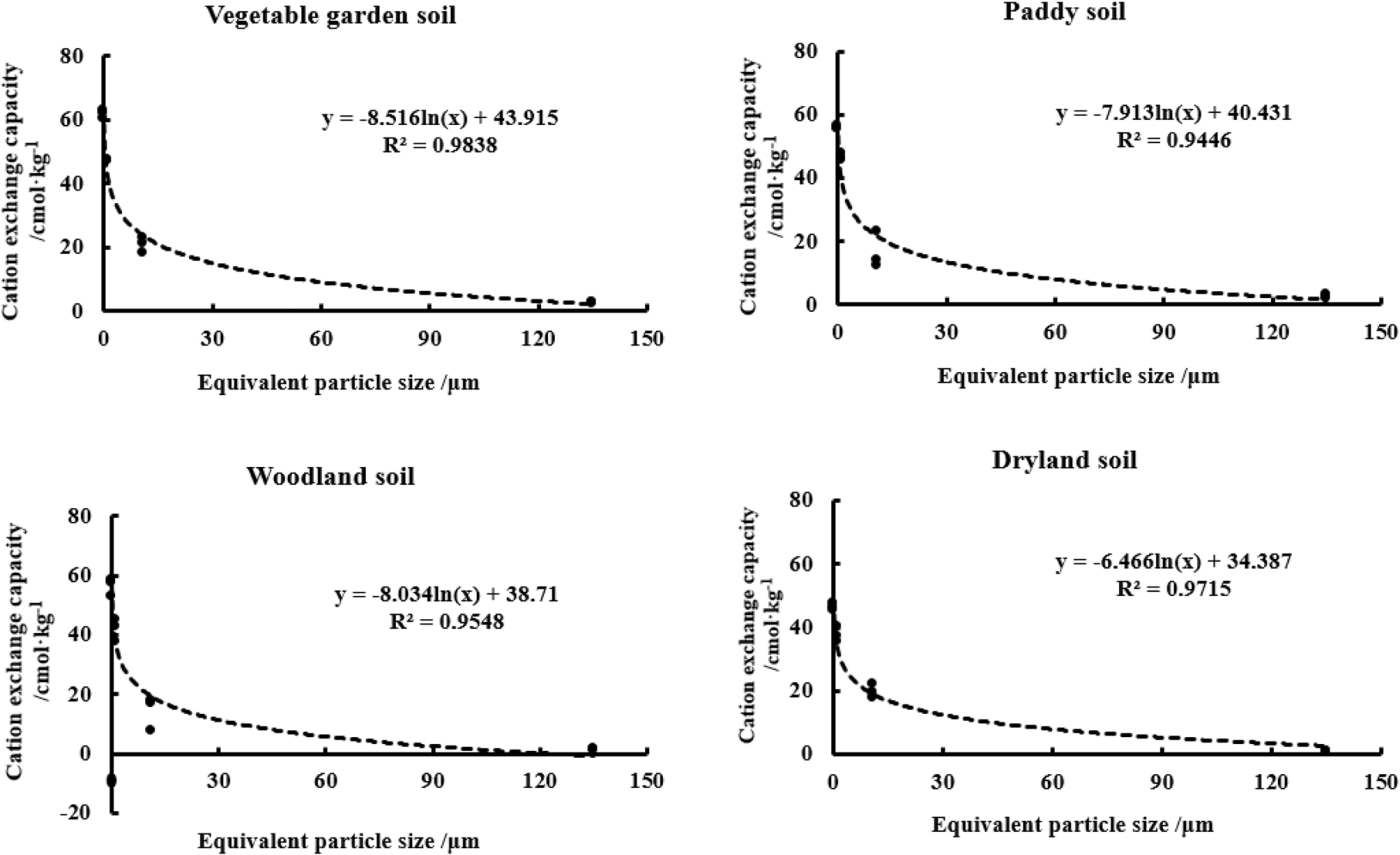
Correlation between CEC and equivalent particle size in yellow–brown soil under different utilizations.

The changes of CEC content in the four soils were shown in Table 5. The CEC content ranges from 0.71 to 23.1cmol·kg^−1^ at the two > 2 μm particle sizes and 37.07–62.91 cmol·kg^−1^ at the two < 2 μm particle sizes. With the grain size getting finer, the CEC content of the soil was gradually increasing, which was in a logarithmic relationship with the equivalent diameter for the four soils with the correlation coefficients (r^2^) of 0.9838, 0.9446, 0.9548, and 0.9715, respectively (n = 12, *p* < 0.01) (Fig. 4).

**Table 5:** Amounts of cation exchange capacity in yellow–brown soils.

Soil layer/cm	Particle size/μm	Equivalent diameter/μm	VG soil		Py soil		WL soil		DL soil		Significant difference between particles
			CEC/cmol·kg^−1^	Relative scale/%	CEC/cmol·kg^−1^	Relative scale/%	CEC/cmol·kg^−1^	Relative scale/%	CEC/cmol·kg^−1^	Relative scale/%	
0–10	20–250	135	2.77	3.8	3.23	5.4	1.27	5.3	0.74	4.6	Dd
	2–20	11	22.7	30.6	23.1	45.8	18.07	28.4	22.12	46.5	Cc
	0.2–2	1.1	47.54	47.3	46.46	36	37.57	50.9	35.67	39.2	Bb
	< 0.2	0.1	62.91	18.2	56.28	12.9	57.77	15.4	45.59	9.7	Aa
	Original soil CEC		21.48		12.93		17.08		9.64		
	Calculated CEC (After particles separation)		24.92		14.26		17.21		10.37		
10–20	20–250	135	2.77	4.8	2.44	4.7	1.29	3.1	0.71	5.2	Dc
	2–20	11	21.28	31.3	14	34.5	17.05	30.9	19.4	30.8	Cc
	0.2–2	1.1	47.38	49.7	47.72	50.1	42.66	56.2	37.07	47.4	Bb
	< 0.2	0.1	60.38	14.1	55.61	10.7	58.69	9.8	47.38	16.5	Aa
	Original soil CEC		19.62		14.02		16.92		9.11		
	Calculated CEC (After particles separation)		22.21		16.18		18.05		9.45		
20–30	20–250	135	2.24	3.4	2.13	6.2	1.69	4.2	0.98	7	Dd
	2–20	11	18.01	26.3	12.23	29.7	7.51	15.2	17.74	20.9	Cc
	0.2–2	1.1	46.96	56.5	45.46	45.2	45.32	65.3	40.23	61.5	Bb
	< 0.2	0.1	61.85	13.9	55.54	18.9	52.81	15.4	46.17	10.7	Aa
	Original soil CEC		20.78		12.37		15.92		8.96		
	Calculated CEC (After particles separation)		22.77		12.92		17.98		9.95

The characteristics of CEC distribution in different profiles of soils with four different utilization methods became smaller overall with the deepening of the soil layer, but the differences were not significant (Fig. 2d). The pH of the soil has some influence on the cation exchange capacity. In general, acidic soils (low pH) lead to a decrease in cation exchange capacity, while alkaline soils (high pH) may increase cation exchange capacity. According to Table 1, there was little variation in pH between the different profiles of the four soils, which explains the insignificant differences in cation exchange. The pH values of the three profiles of the dry farmland were lower than those of the other three soils, and the dry farmland formed a sandy soil with a smaller specific surface area due to its low water content, resulting in a relatively low cation exchange capacity. Therefore, its cation exchange capacity is the weakest and lowest among the four soils. Besides, the high organic matter content may also cause the possible reasons for the high cation exchange, and organic matter plays an important role in the ion exchange of the soil. Soils enriched in organic matter usually have a higher cation exchange capacity because the negative ions in organic matter can form complexes with cations, as in the case of vegetable garden plots. Finally, fine-grained soils (e.g. woodland soils) have a higher specific surface area and ion exchange capacity because their fine particles have more surface available for ion adsorption. Paddy soils are usually in a moist state and rice roots are more active, which helps to promote ion release and exchange. This may lead to an increase in cation exchange.

For the four soils, the differences in CEC content among the four particle sizes of the same soil layer reached an extremely significant level. The difference in CEC content in different soil layers of the same soil is not significant. Therefore, soil particle size has a great influence on CEC content, while soil depth has no obvious influence on CEC content. By calculation, CEC content after particle separation, CEC calculated by different particle sizes is about 4–16% higher than CEC of original soil. This may be because after classifying particles with different particle sizes, some points covered by organic matter are exposed, which may increase the amount of negative charge of the soil.

The CEC relative scale of the four soils in different particle sizes showed that 0.2–2 μm (36.0–65.3%) > 2–20 μm (15.2–46.5%) > less than 0.2 μm (9.7–18.9%) > 20–250 μm (3.1–7.0%) particles. In the yellow–brown soil with four different utilizations, although the CEC of clay particles was the largest, the content of clay particles in the soil was relatively low, resulting in the CEC relatively low proportion. Similarly, the CEC of the 2-20 μm particles was not very high, due to the particles high content in the soil, resulting in the SSA relatively higher proportion.

### Relationship between SSA and OC (N) content, between CEC and OC (N) content, between CEC and SSA in yellow–brown soil under different utilizations

**Figure 5:**
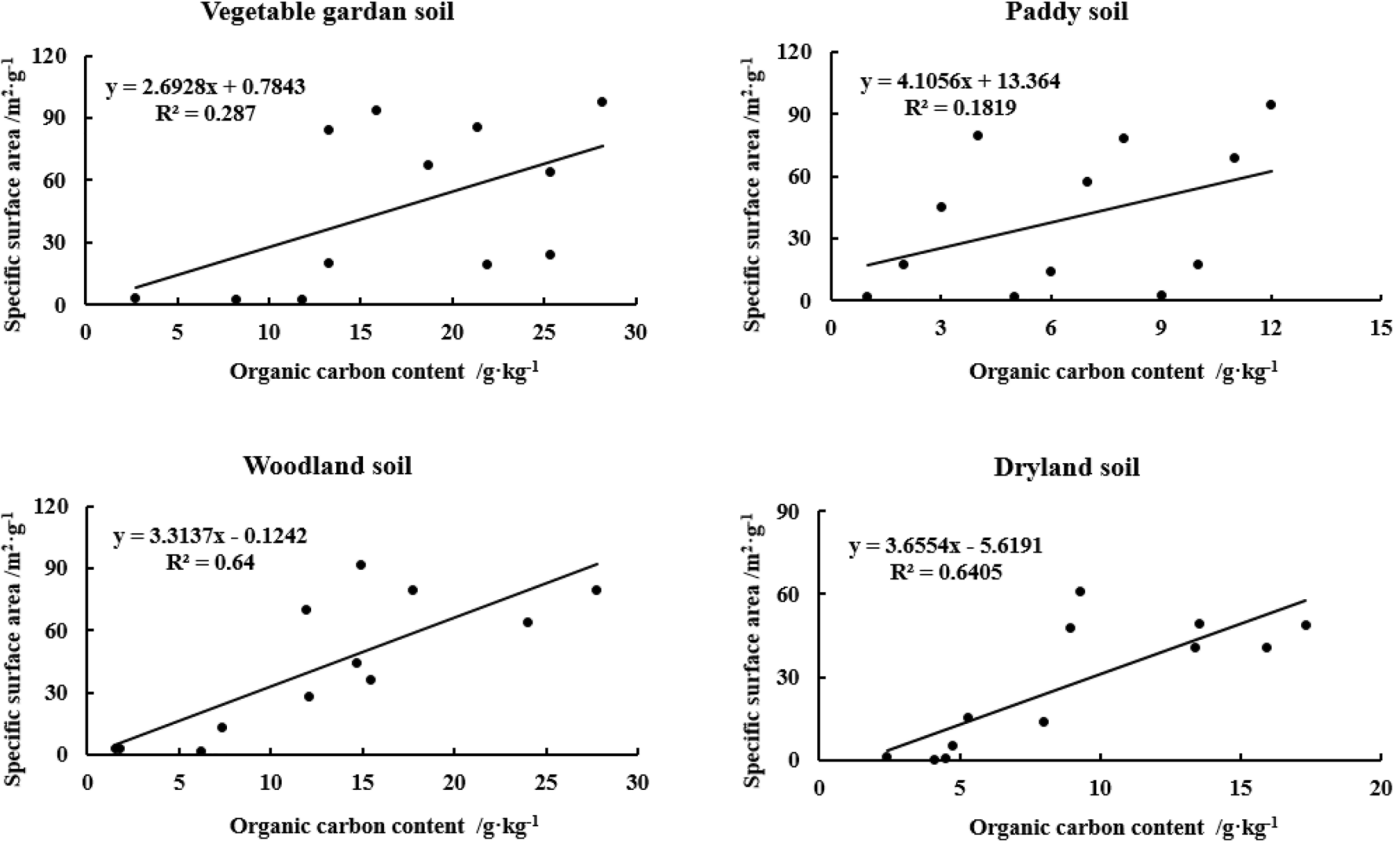
Correlation between SSA and OC content in different particle sizes of yellow–brown soil under different utilizations.

**Figure 6:**
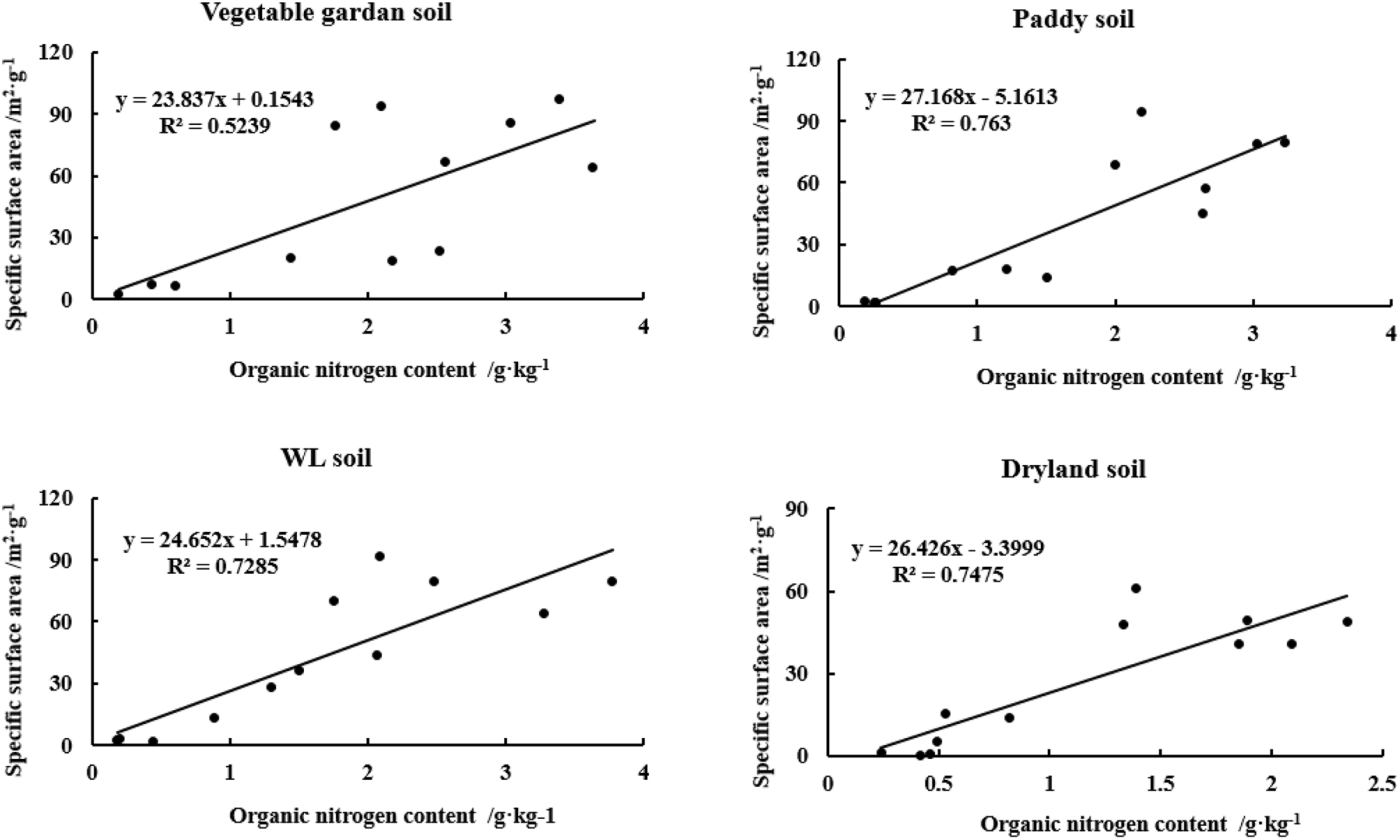
Correlation between SSA and ON content in different particle sizes of yellow–brown soil under different utilizations.

The relationship between SSA and OC content of yellow–brown soils under different utilizations showed that the linear correlation between SSA and OC of woodland soil (r^2^, 0.6400) and dryland soil (r^2^, 0.6405) was significantly higher than that of vegetable garden soil (r^2^, 0.2870) and paddy soil (r^2^, 0.1819) (Fig. 5). However, there were good linear relationships between SSA and ON for the four soils, with the correlation coefficients of 0.5239, 0.763, 0.7285 and 0.7475, respectively, and the linear relationship reached a very significant level (n = 12, *p* < 0.01) (Fig. 6).

Most surface phenomena are related to SSA. According to the experimental results, most soil OC and ON are concentrated in clay. Clay contains a large amount of Fe-Al oxide, which has a huge surface area and is the material basis for preserving soil OC and nitrogen^22,27,46^. A notable phenomenon is that the content of organic matter decreases while the SSA increases at the depth of 20–30 cm section, which may indicate that the coverage of OC on the mineral surface affects the results of SSA measurement. This may be related to the method of measuring SSA. The surface area is measured by the N_2_ adsorption method. Organic matter covers the surface of clay minerals, and 95–99% of the surface area of soil organic matter is distributed in the pore size of < 0.5 nm. This pore size limits the diffusion of N_2_ molecules^47^, affects the diffusion of N_2_ into the small pores of micro-aggregates, and may underestimate the surface area of soil with higher organic matter^10,14,15^.

**Figure 7:**
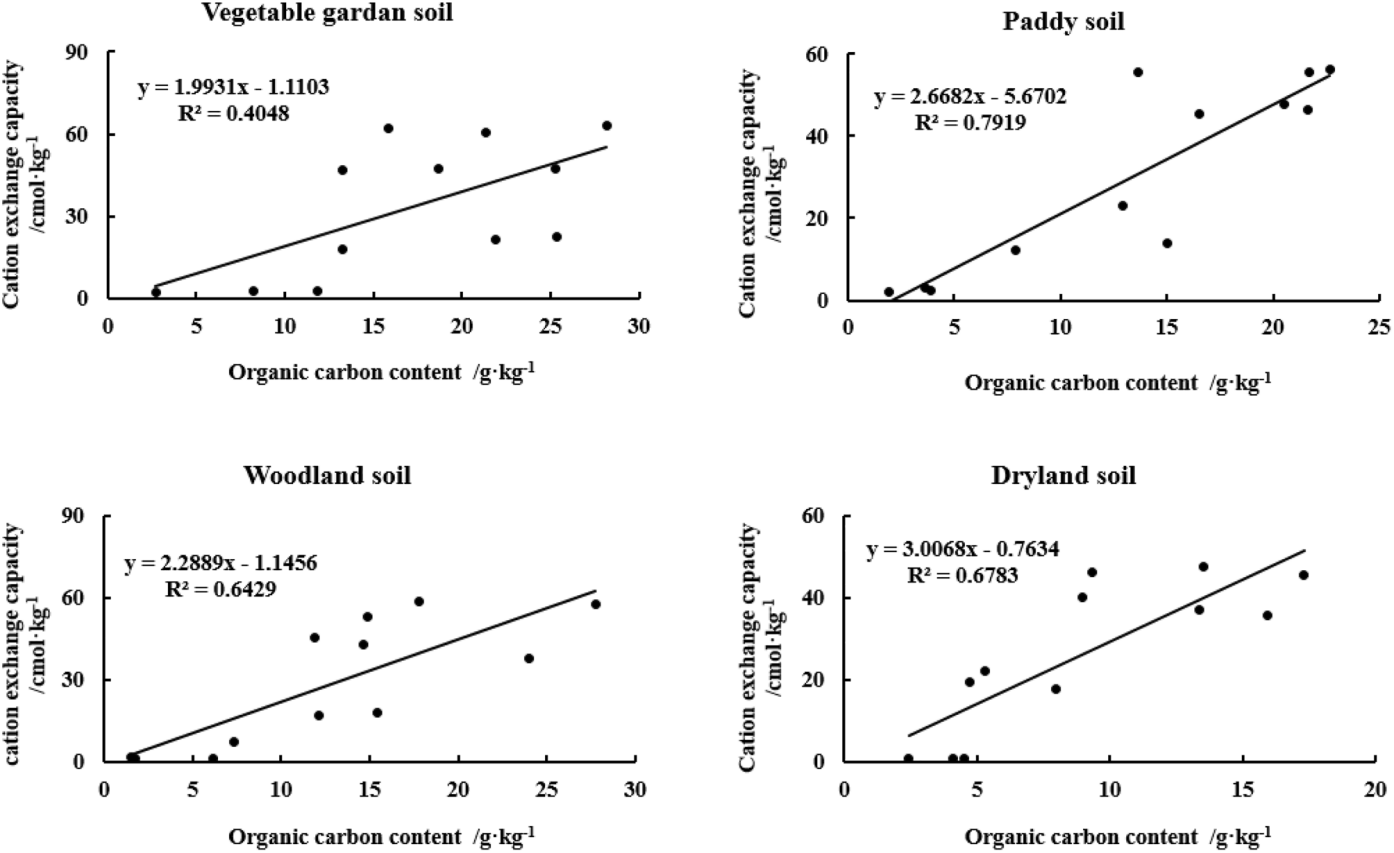
Correlation between CEC and OC content in different particle sizes of yellow–brown soil under different utilizations.

**Figure 8:**
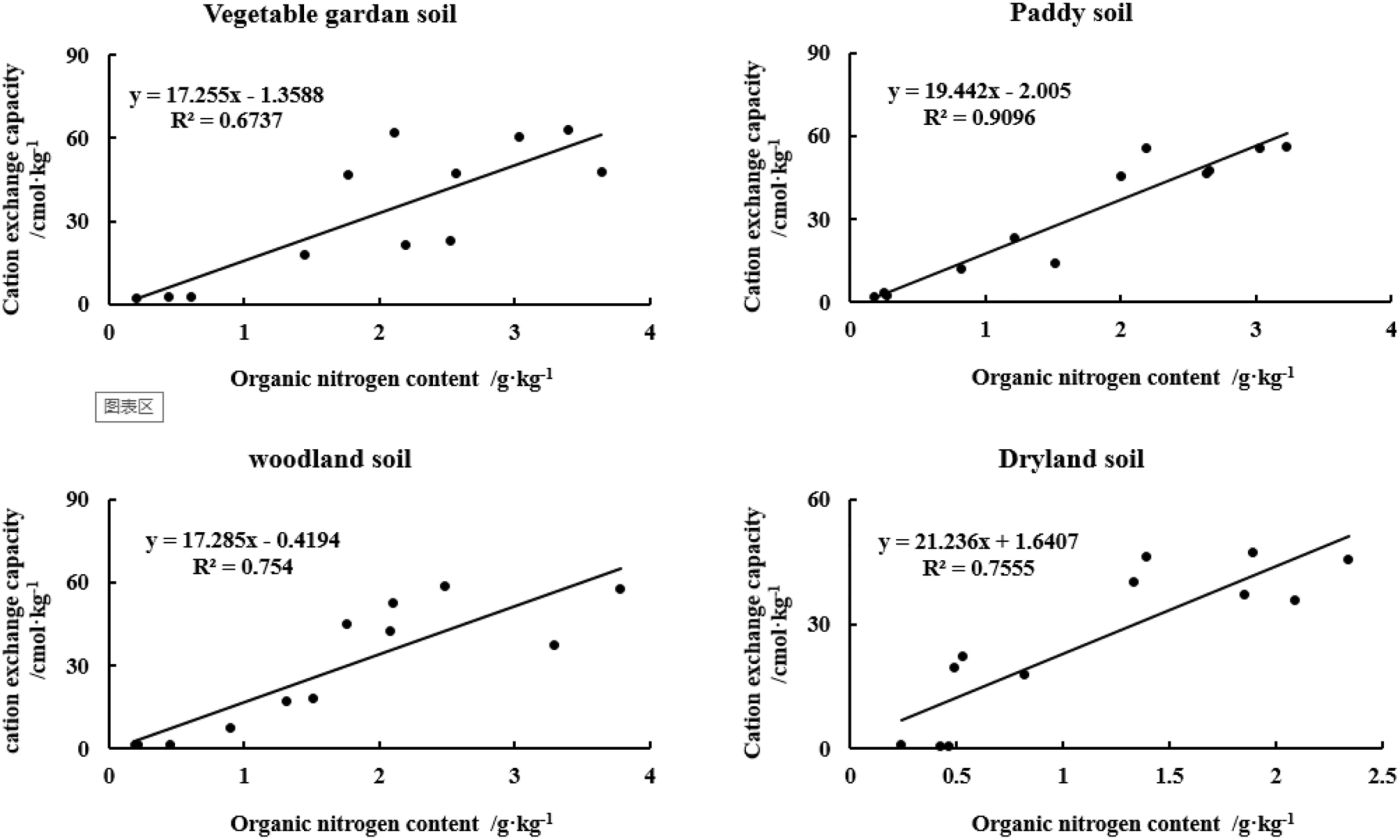
Correlation between CEC and ON content in different particle sizes of yellow–brown soil under different utilizations.

The key indicators were determined based on the correlation between the CEC and OC (N) contents of the yellow–brown soils under different utilization conditions. The CEC of all three soils, paddy, woodland, and dryland soils, showed a significant positive linear correlation with OC content (n = 12, *p* < 0.01) (Fig. 7). Among them, the effect of OC on CEC was slightly less significant for the vegetable garden soil than for the other three soils. As for CEC and ON, the linear correlation systems between ON and CEC content in all four soils reached a highly significant level, with the linear correlation coefficients (r^2^) in vegetable garden soil, paddy soil, woodland soil, and dry land soil were 0.6737, 0.9096, 0.7540, and 0.7555, respectively (n = 12, *p* < 0.01) (Fig. 8).

Soil CEC depends not only on the quantity and composition of clay minerals but also on the quantity of organic matter, which reflects the negative point of clay minerals and organic matter^22,48^. According to the experimental results, the linear correlation coefficients (r^2^) between OC and CEC in paddy soil, woodland soil, and dryland soil are 0.7919, 0.6429, and 0.6783(n = 16, *p* < 0.01), which are extremely significant. However, the linear correlation coefficient between cation substitution and OC in vegetable garden soil is only 0.4048(n = 16, *p* < 0.05), which is also significant. The linear correlation coefficients (r^2^) between ON and CEC in vegetable garden soil, paddy soil, woodland soil, and dry land soil were 0.6737, 0.9096, 0.754, and 0.7555(n = 16, *p* < 0.01), respectively, which were extremely significant. Sposito^49^ studied 3,000 mineral soils, which showed that there was a significant relationship between soil OC and CEC content. Kaiser et al.^22^ extracted OC with pyrophosphate, accounting for 13–34% of the total OC in soil. They believed that CEC was closely related to the content of clay (r^2^ = 0.95) and OC (r^2^ = 0.71). Perhaps CEC was more suitable for describing the variable of soil OC capacity. Hepper et al.^48^ studied the volcanic ash soil rich in illite and found that CEC was positively correlated with soil OC (r^2^ = 0.837, n = 9). The cation exchange depends on the SSA and the number and position of negative charge exchange points on the particle surface^4^, and OC and ON greatly affect the cation content^4–6^.

**Figure 9:**
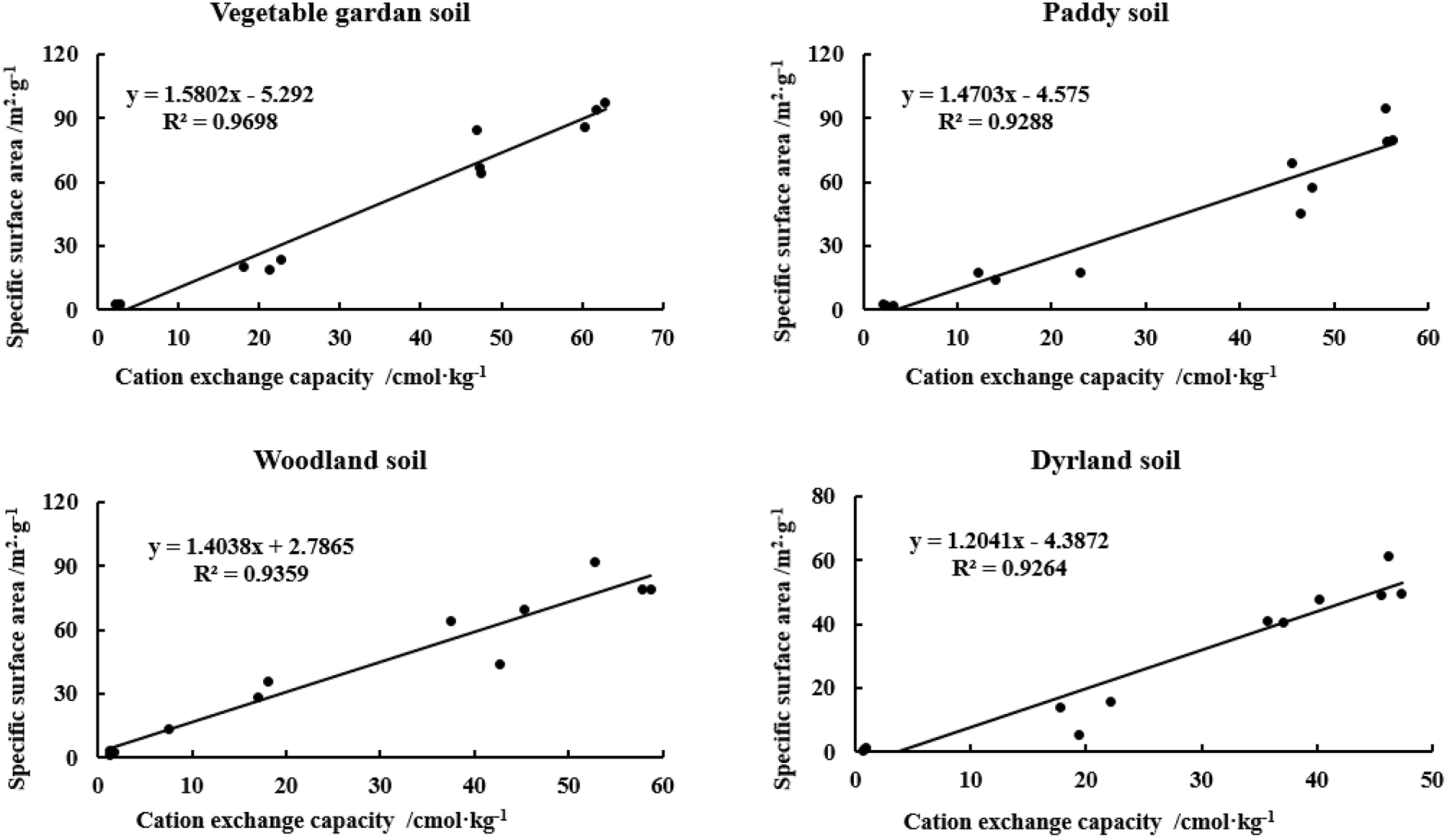
Relationship between CEC and SSA in different particle sizes of yellow–brown soil under different utilizations.

To verify the correlation between CEC and SSA in different particle sizes of yellow–brown soils under different utilization conditions, we plotted the relationship between CEC and SSA of four soils (Fig. 9). From the figure, it was obvious that the CEC of all four soils showed an extremely significant linear relationship with SSA, and the correlation coefficients r^2^ of vegetable garden soil, paddy soil, woodland soil, and dryland soil were 0.9698, 0.9289, 0.9359, and 0.9264, respectively (n = 12, *p* < 0.01).

The correlation coefficients between CEC and SSA of vegetable garden soil, paddy soil, woodland soil, and dryland soil are 0.9698, 0.9288, 0.9359, and 0.9264 (n = 16, *p* < 0.01), respectively, and CEC and SSA of the four soils are extremely significant. With the increase of SSA, CEC increases linearly. The influence of micro-aggregates on CEC is more complicated than that of SSA because the amount of cation substitution depends on SSA and cation substitution sites on the surface^50,51^. Kahle et al.^51^ believe that CEC and SSA have an extremely significant positive linear correlation, and Ersahin et al.^50^ also believe that CEC and SSA have an extremely significant linear correlation, with a correlation coefficient of 0.98. Theng et al.^52^ believe there is a good correlation between the SSA of soil measured by N_2_O and CEC. Because the formation of the organic-mineral complex is influenced by a cationic bond bridge, NH_4_^+^ can freely diffuse into the micropores of organic matter and clay, and the contribution of clay and organic matter to the negative charge points of CEC is almost independent. However, Hepper et al.^48^ thought that the linear relationship between CEC and SSA was not significant (R^2^ = 0.175, *p* < 0.05, n = 24) in soils with high organic matter content and expansive minerals. The cation exchange capacity depends on the inner and outer surface properties of minerals, while the SSA measured by the N_2_ gas adsorption method is limited to the outer surface.

## Conclusion

1. The size of soil particles has a great influence on the content of OC(N) in yellow–brown soil. Soil particle size distribution is closely related to soil organic carbon content, and has a significant impact on soil organic carbon conversion. With the grain size becoming finer, Soil OC(N) generally increased gradually. The difference in soil OC(N) content between < 2 μm and 2-20 μm and 20-250 μm was extremely significant. The C/N ratio of > 2 μm two particles was higher than < 2 μm two particles and the former was significantly different from the latter (*p* < 0.05). There were good linear relationships between OC and ON in different particle sizes of yellow–brown soil under different utilizations and the linear relationship reached a very significant level (r^2^ > 0.86) (n = 12, *p* < 0.01).
2. Both the SSA and CEC increase with the decrease in particle size. Grain size has a significant effect on SSA and CEC content in yellow–brown soil. There were logarithmic relationships between SSA and the equivalent diameter (r^2^ > 0.91) and between CEC and the equivalent diameter (r^2^ > 0.94) for the four soils (n = 12, *p* < 0.01).
3. The linear correlation between SSA and OC of woodland soil and dryland soil was significantly higher than that of vegetable garden soil and paddy soil. However, the linear relationships between SSA and ON for the four soils all reached a very significant level (r^2^ > 0.52) (n = 12, *p* < 0.01). The CEC of four soils showed a significant linear correlation with OC content (r^2^ > 0.40) and ON content (r^2^ > 0.67) (n = 12, *p* < 0.01). Moreover, there was an extremely significant linear relationship between CEC and SSA of the four soils (r^2^ > 0.92) (n = 12, *p* < 0.01).
4. Management: Consider managing soil particle size to optimize OC(N) content. Finer particle sizes generally lead to increased OC(N) content, so practices such as appropriate tillage, organic amendments, or cover cropping can promote the accumulation of organic matter in finer soil fractions. Tailor fertilizer application strategies based on particle size and associated nutrient dynamics. Adjusting nutrient ratios based on specific particle size fractions can help address nutrient imbalances and promote efficient nutrient utilization, contributing to improved soil fertility and sustainable agricultural production. Recognize that the relationships between SSA, CEC, OC(N), and particle size can vary with different land-use practices. The results showed that increasing the content of sand and silt in the topsoil, returning farmland to forest and planting in wasteland could improve soil quality, restore soil fertility and increase carbon storage, which was the fundamental guarantee for the restoration and reconstruction of vegetation in this region. Therefore, when designing land-use systems, take into account the specific soil types and their relationships between these properties to ensure appropriate management and maintenance of soil fertility.

## Data Availability

All data that support the findings of this study are available from the corresponding author on request.
